# Similar secondary patellar resurfacing but higher tibial revision risk with posterior stabilized versus medial pivot total knee arthroplasty: A registry analysis

**DOI:** 10.1002/jeo2.70751

**Published:** 2026-05-20

**Authors:** Patrick Pflüger, Alberto Pedrazzini, Lukas Jud, Lazaros Vlachopoulos, Sandro F. Fucentese

**Affiliations:** ^1^ Department of Orthopedics Balgrist University Hospital University of Zurich Zurich Switzerland

**Keywords:** complications, joint registry, knee osteoarthritis, revision, total knee arthroplasty

## Abstract

**Purpose:**

Patellar complications are among the most frequent causes of revision after primary total knee arthroplasty (TKA). The primary study objective is to compare the rate of secondary patellar resurfacing between medial pivot (MP) and posterior stabilized (PS) designs in TKAs.

**Methods:**

Analysis of the Swiss national knee joint registry, including TKAs without patellar resurfacing at index surgery performed between 2012 and 2023. Only TKAs with MP and PS designs featuring cemented femoral and tibial components were included. Time‐to‐event analysis was performed using Kaplan–Meier curves and Cox proportional hazards regression. Multivariable models adjusted for age, sex and BMI.

**Results:**

A total of 32,540 TKAs were included in the final analysis. Component revision was performed in 5.8% (*n* = 1873) of cases. The revision rate was 5.2% (*n* = 916) for MP and 6.4% (*n* = 957) for PS designs (*p* = 0.7). Secondary patellar resurfacing was the most common revision procedure, accounting for 41.8% (*n* = 383) of MP revisions and 37.7% (*n* = 361) of PS revisions. No significant difference was observed between designs for secondary patellar resurfacing (adjusted hazard ratio [HR] = 0.93; 95% confidence interval [CI] = 0.79–1.11; *p* = 0.43). PS designs showed significantly higher risk for tibial revision (unadjusted HR = 1.55; 95% CI = 1.07–2.25; *p* = 0.02, adjusted HR = 1.51; 95% CI = 0.98–2.31; *p* = 0.06). Younger age and female sex were independent risk factors for both revision types.

**Conclusion:**

No difference in secondary patellar resurfacing rates was observed between MP and PS designs in primary TKA. However, PS designs are associated with increased tibial revision risk compared to MP designs.

**Level of Evidence:**

Level III.

**Abbreviations**
ANQ: National Association for Quality Control in Hospitals and Clinics in Switzerland
ASA: American Society of Anesthesiologists
BMI: body mass index
CI: confidence interval
CR: cruciate retaining
HR: hazard ratio
IQR: interquartile range
ISAR: International Society of Arthroplasty Registries
MP: medial pivot
OA: osteoarthritis
PE: polyethylene
PROM: patient‐reported outcome measure
PS: posterior stabilized
Q‐Q: quantile–quantile
SD: standard deviation
SIRIS: Swiss Implant Registry for Knee and Hip
TKA: total knee arthroplasty

## INTRODUCTION

Patellar complications constitute the leading cause of revision surgery following primary total knee arthroplasty (TKA), with secondary patellar resurfacing without additional procedures accounting for up to one‐quarter of all interventions within 2 years of the index surgery [2, 26].

Registry data reveal substantial international variation in secondary patellar resurfacing rates, influenced by geographical differences, implant selection and design characteristics [2, 10, 26]. Notably, the German arthroplasty registry documented significant design‐dependent disparities, with the posterior stabilized (PS) Vega prosthesis demonstrating markedly higher secondary patellar resurfacing rates compared to the cruciate retaining (CR) Columbus system [3, 10].

The aetiology of patellar complications is multifactorial, encompassing patient‐specific characteristics, surgical techniques and implant design features [20]. Furthermore, primary patellar resurfacing does not universally confer superior clinical outcomes and carries a non‐negligible complication risk of up to 7%, supporting a selective rather than systematic resurfacing strategy [7, 24].

Biomechanical analyses have elucidated distinct patellofemoral kinematic patterns across different prosthetic designs. Comparative studies between CR and PS TKAs have shown significant alterations in patellofemoral mechanics [3]. Similarly, analyses comparing medial pivot (MP) and PS designs have identified notable kinematic variation [4, 11]. These biomechanical differences suggest potential clinical advantages for MP designs in mitigating patellofemoral complications compared to PS systems [11, 13, 23]. However, meta‐analyses reported no difference in complications between MP and PS designs [12, 14]. The Australian registry shows lower overall revision rates for MP designs compared to PS systems, though revision‐specific data are not stratified [2]. Furthermore, the German registry demonstrates marked heterogeneity in secondary patellar resurfacing rates across different implant systems [10].

Beyond patellofemoral complications, TKA design appears to influence overall long‐term survival. Meta‐analyses and registry data suggest potential superiority of CR compared to PS TKAs [2, 10, 15, 28]. Notably, aseptic loosening occurs more frequently in PS compared to CR TKAs, affecting both tibial and femoral components [15, 28].

This study aims to systematically analyze the rate of secondary patellar resurfacing following primary TKA. Based on existing biomechanical evidence, clinical observations and international registry findings, we hypothesize that the rate of secondary patellar resurfacing is lower following MP in comparison to PS TKAs. Secondarily, we analyzed the femoral and tibial revision rates between these two TKA designs.

## METHODS

This is an analysis of the nationwide knee joint registry in Switzerland (SIRIS). The registry was mandated by the National Association for Quality Control in Hospitals and Clinics in Switzerland (ANQ) and introduced in 2012. All hospitals and clinics that have joined the ANQ National Quality Contract and perform implantations in the areas defined by the ANQ are obligated to register. The SIRIS hip and knee registry achieves a registration rate of 98%, which exceeds the required standard of the International Society of Arthroplasty Registries (ISAR) for full membership. Annual reports of the SIRIS are published to provide an overview of the development of the implanted hip and knee arthroplasties [26].

Third parties can submit an application for data analysis of a specific research question.

Using electronic data collection forms, the following data are collected for the implantation of a primary TKA:

- Demographics
- Clinic, surgeon, surgery date
- Risk factors (American Society of Anesthesiologists [ASA] and body mass index)
- Primary pathology (diagnosis and previous surgeries)
- Procedure (date, side and procedure type)
- Technology (conventional, minimally invasive, computer‐assisted and patient‐specific)
- Component fixation (fixation type, cementing technique and cement)
- Implant data

### Study cohort

**Figure 1:**
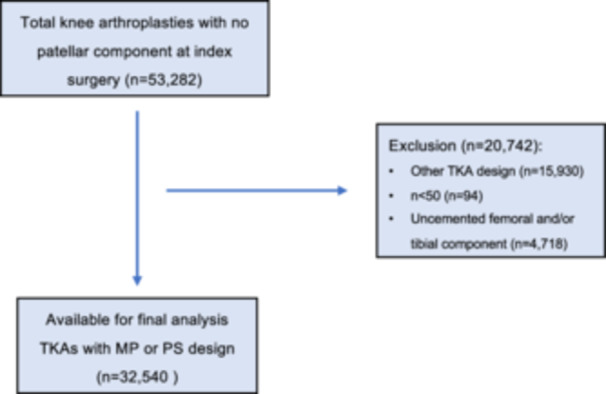
Flowchart depicting the inclusion/exclusion of total knee arthroplasties. MP, medial pivot; PS, posterior stabilized; TKA, total knee arthroplasty.

Following our application for data analysis of the SIRIS knee registry, only primary TKAs without patellar resurfacing at index surgery were included. According to the implant/inlay design, TKAs were stratified into MP and PS. Only MP and PS TKAs with more than 50 registered cases were considered. To omit potential biases regarding the revision, only TKAs with cemented femoral and cemented tibial components were included for the final analysis (Figure 1).

The baseline data of the included patients stratified according to implant type/design are illustrated in Table 1.

**Table 1:** Baseline data of the included patients, stratified according to medial pivot and posterior stabilized TKAs.

	Medial pivot (*N* = 17,579)	Posterior stabilized (*N* = 14,961)	*p*
Age (years) at index surgery (mean ± SD)	69.1 ± 9.5	69.7 ± 9.6	<0.001
Sex (female)	58.2% (*n* = 10,232)	59.6% (n = 8,919)	0.01
BMI (kg/m^2^)	28.7 (7.3) (*n* = 15,018)	29 (7.4) (*n *= 9,823)	<0.001
Primary OA	89.1% (*n* = 15,659)	88.7% (*n* = 13,275)	0.33

Abbreviations: BMI, body mass index; OA, osteoarthritis; SD, standard deviation; TKA, total knee arthroplasty.

### Data Analyses

Continuous variables are presented as mean ± standard deviation (SD) or median with interquartile range (IQR), as appropriate. Categorical variables are presented as frequencies and percentages. RStudio (R version 4.4.0 (2024‐04‐24), PBC) was used for data processing, and a *p* value < 0.05 was considered statistically significant.

Data normality was assessed graphically (quantile–quantile [Q–Q] plot) and numerically (Shapiro–Wilk test).

Time‐to‐event analysis was conducted using the Kaplan–Meier method to estimate survival probabilities and generate survival curves. The primary endpoint was time to revision (secondary patellar resurfacing, femoral and tibial revision), defined as the time interval from the index surgery to the occurrence of the event of interest. Patients without events were censored at the date of last follow‐up or death, whichever occurred first.

Kaplan–Meier survival curves were constructed to estimate event‐free survival probabilities by prosthesis design (MP vs. PS) and compared using the log‐rank test to assess for statistically significant differences in survival distributions. Survival estimates with 95% confidence intervals (CIs) were calculated at 2‐ and 5‐year post‐surgery, along with the number of patients at risk at these time points. Both unadjusted and adjusted Cox proportional hazards models were fitted to estimate hazard ratios (HRs) comparing MP to PS designs. The unadjusted model included prosthesis design as the sole predictor. The adjusted model incorporated the following covariates based on clinical relevance and potential confounding: age, sex and BMI. Missing BMI data were handled using mean imputation (primary analysis) and complete case analysis (sensitivity analysis).

## RESULTS

In total, 1873 patients underwent a revision of the component (Table 2). The overall component revision rate was not statistically significantly different between MP and PS TKAs. Only tibial revisions occurred significantly more in PS compared to MP TKAs (*p* = 0.046) (Table 2).

**Table 2:** Overview of all component revisions performed stratified according to the design of the implant and cause of revision.

	Medial pivot (*n* = 17,579)	Posterior stabilized (*N* = 14,961)	*p*
Revision	5.2% (*n* = 916)	6.4% (*n* = 957)	0.7
Cause for revision			
Secondary patella resurfacing	41.8% (*n* = 383)	37.7% (*n* = 361)	0.25
Patella revision without resurfacing	5.2% (*n* = 48)	6.2% (*n* = 59)	0.47
Complete revision	24.0% (*n* = 220)	25.9% (*n* = 248)	0.5
Component removal with/without spacer	3.5% (*n* = 32)	2.4% (*n* = 23)	0.22
Femoral revision	1.9% (*n* = 17)	2.3% (*n* = 22)	0.62
Tibial revision	5.0% (*n* = 46)	7.5% (*n* = 72)	0.046*
Only change of PE Inlays	15.3% (*n* = 140)	14.7% (*n* = 141)	0.82
Other	1.4% (*n* = 13)	1.6% (*n* = 15)	0.94
Reimplantation of prosthesis	1.6% (*n* = 15)	1.7% (*n* = 16)	1.0
Arthrodesis	0.2% (*n* = 2)	0% (*n* = 0)	0.46

Abbreviation: PE, polyethylene.

*Statistically significant.

### Secondary patellar resurfacing

**Figure 2:**
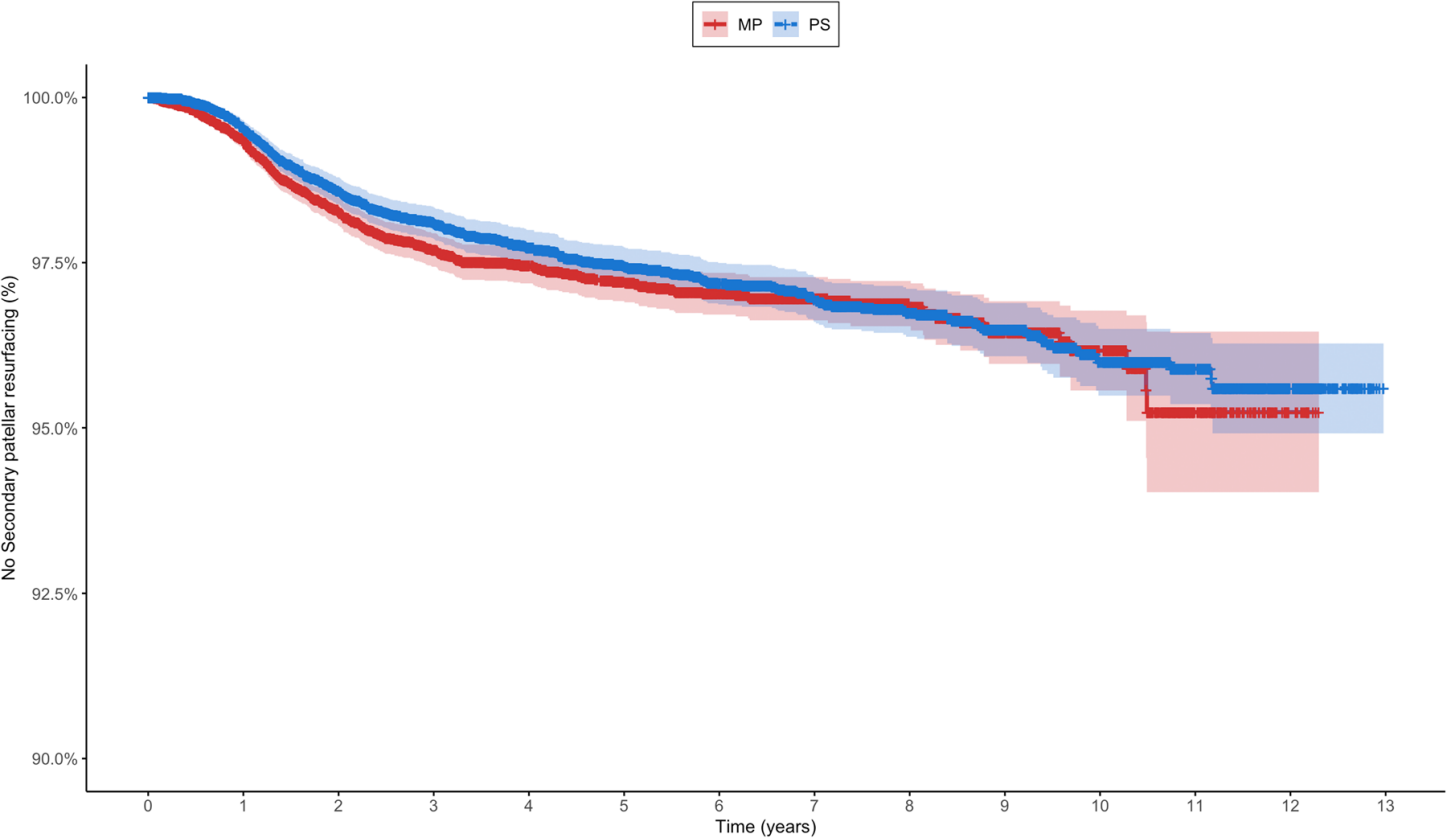
Kaplan–Meier survival curves for secondary patellar resurfacing comparing MP and PS TKAs. MP, medial pivot; PS, posterior stabilized; TKA, total knee arthroplasty.

Among 32,540 patients, 744 (2.29%) underwent secondary patellar resurfacing: 383/17,579 (2.18%) with MP designs and 361/14,961 (2.41%) with PS designs (Table 3, Figure 2). Neither unadjusted (HR = 0.91; 95% CI: 0.79–1.06; *p* = 0.22) nor adjusted analysis (HR = 0.93; 95% CI: 0.79–1.11; *p* = 0.43) revealed significant differences between implant types. Younger patients were significantly more likely to require secondary patellar resurfacing, with each additional year of age reducing the risk by 3% (HR = 0.97 per year; *p* < 0.001). Female sex was also an independent risk factor (HR = 1.21; *p* = 0.04).

**Table 3:** Kaplan–Meier survival analysis for secondary patellar resurfacing.

Type	2‐year survival with 95% CI	Number at risk at 2 years	5‐year survival with 95% CI	Number at risk at 5 years
Medial pivot	98.3% (98.1%–98.5%)	12.009	97.2% (96.9%–97.5%)	5.762
Posterior stabilized	98.6% (98.4%–98.8%)	11.650	97.4% (97.2%–97.7%)	7.500

Abbreviation: CI, confidence interval.

### Femoral and tibial revisions

**Figure 3:**
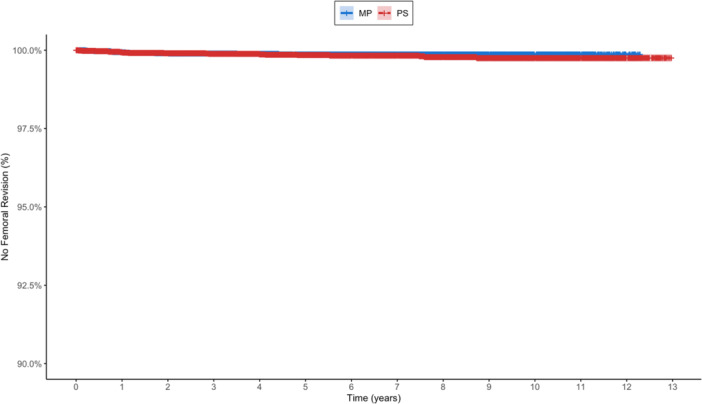
Kaplan–Meier survival curves for femoral revisions comparing MP and PS TKAs. MP, medial pivot; PS, posterior stabilized; TKA, total knee arthroplasty.

Femoral revision was documented in 39 out of 32,540 patients (0.12%), comprising 17/17,579 (0.097%) MP cases and 22/14,961 (0.147%) PS cases (Table 4, Figure 3). Unadjusted analysis revealed no significant difference in femoral revision hazard between design types (HR = 1.30; 95% CI: 0.69–2.46; *p* = 0.42). This finding persisted in adjusted models, whether using imputed BMI data (HR = 1.34; 95% CI: 0.71–2.53; *p* = 0.37; *n* = 32,535) or restricting to complete cases (HR = 1.28; 95% CI: 0.60–2.74; *p* = 0.52). Decreasing age was the only factor significantly associated with higher femoral revision risk (HR = 0.96 per year; *p* = 0.026), with sex and BMI showing no meaningful associations.

**Table 4:** Kaplan–Meier survival analysis for femoral revisions.

Type	2‐year survival with 95% CI	Number at risk at 2 years	5‐year survival with 95% CI	Number at risk at 5 years
Medial pivot	99.9% (99.9%–100%)	12.009	99.9% (99.8%–99.9%)	5.762
Posterior stabilized	99.9% (99.9%–100%)	11.650	99.8% (99.8%–99.9%)	7.500

Abbreviation: CI, confidence interval.

**Figure 4:**
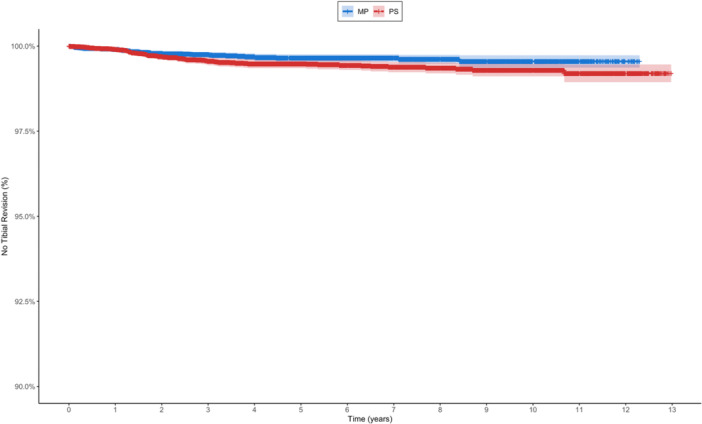
Kaplan–Meier survival curves for tibial revisions comparing MP and PS TKAs. MP, medial pivot; PS, posterior stabilized; TKA, total knee arthroplasty.

Among 32,540 patients, 118 (0.36%) underwent tibial revision: 46/17,579 (0.26%) with MP designs and 72/14,961 (0.48%) with PS designs (Table 5, Figure 4). Unadjusted analysis revealed significantly higher tibial revision risk with PS designs (HR = 1.55; 95% CI: 1.07–2.25; *p* = 0.020). In multivariable analysis using BMI imputation (*n* = 32,535), this association remained significant (HR = 1.57; 95% CI: 1.08–2.28; *p* = 0.02), while complete case analysis showed a similar but marginally non‐significant trend (HR = 1.51; *p* = 0.06). Additional risk factors included younger age (HR = 0.97 per year; *p* = 0.002) and female sex (HR = 1.54; *p* = 0.031).

**Table 5:** Kaplan–Meier survival analysis for tibial revisions.

Type	2‐year survival with 95% CI	Number at risk at 2 years	5‐year survival with 95% CI	Number at risk at 5 years
Medial pivot	99.8% (99.7%–99.9%)	12.009	99.7% (99.5%–99.8%)	5.762
Posterior stabilized	99.7% (99.6%–99.8%)	11.650	99.5% (99.4%–99.6%)	7.500

Abbreviation: CI, confidence interval.

## DISCUSSION

The most important finding of this study is that there was no significant difference in survival regarding secondary patellar resurfacing between MP and PS designs in primary TKA. Therefore, our hypothesis that the rate of secondary patellar resurfacing is lower following MP in comparison to PS TKAs was rejected.

### Secondary patellar resurfacing

Registry data revealed significant design‐dependent disparities in regard to secondary patellar resurfacing rates [3, 10, 21]. This data was further supported by comparative biomechanical analysis, which demonstrated notable differences in patellofemoral mechanics between CR and PS TKAs [3]. Comparing MP and PS TKAs, biomechanical studies revealed design‐dependent differences regarding the change of patellofemoral kinematics and retropatellar pressure in comparison to the native knee [11]. The MP design showed an improved quadriceps efficiency in comparison to CR TKA [4]. These biomechanical properties of MP designs might reduce patellofemoral‐related complications in comparison to PS systems [13]. However, meta‐analyses investigating the clinical outcomes (range of motion), patient‐reported outcomes (PROMs) and complications (stiffness, aseptic revision) reported no differences between MP and PS TKAs [12, 14]. None of the previously referenced meta‐analyses investigated the rate of secondary patellar resurfacing between MP and PS designs. Other registry data showed relevant heterogeneity with regard to implants and their rate of secondary patellar resurfacing, or do not further stratify the type of secondary procedure performed for the implant types [2, 10]. Our study, therefore, provides a detailed analysis of the rate of secondary patellar resurfacing between MP and PS TKAs. The SIRIS knee registry showed that there was no significant difference in survival regarding secondary patellar resurfacing between cemented, cruciate‐sacrificing MP and PS TKAs. This might be attributed to the low event rate of 2.29%, despite being the most commonly performed implant revision following primary TKA.

Current research approaches face inherent limitations that prevent comprehensive risk factor analysis. Due to their large patient cohorts, registry studies can provide robust analyses for identifying patient‐related and implant‐related risk factors [21]. However, radiographic and morphologic parameters, alignment philosophy, and its influence on trochlear engagement and patellofemoral kinematics are not captured in registry data, and are typically investigated in clinical studies that often include substantially fewer patients [8, 19, 22, 27]. Given the relatively low event rate of secondary patellar resurfacing, these smaller clinical studies lack sufficient statistical power to perform meaningful multivariate analyses incorporating all potential risk factors.

A combination of different datasets might help to further understand the potential risk factors and their clinical significance. The management of patellar resurfacing in TKA remains highly controversial, with practice varying substantially between surgeons and institutions based on training background, geographic patterns and hospital policy rather than clinical criteria alone [5, 16]. Given that anterior knee pain represents a significant cause of patient dissatisfaction after TKA, future investigations should assess the relative importance of different risk factors and their interactions [17, 27].

### Femoral and tibial revision

Implant design influences revision risk in published literature, yet our analysis revealed no differences in overall component revision rates between MP and PS designs. A meta‐analysis of seven studies with a minimum 10‐year follow‐up reported lower rates of aseptic loosening in CR compared to PS TKAs, though the anatomical location of loosening was not specified in the included studies [1, 15, 18]. Similarly, the Australian Orthopaedic Association National Joint Replacement Registry documented differences in loosening rates between PS and minimally stabilized designs [28]. Registry data from Germany confirm that loosening represents the most common indication for TKA revision, with tibial component loosening occurring more frequently than femoral loosening, and PS designs demonstrating higher cumulative failure rates compared to CR designs [10].

Despite similar overall revision rates, component‐specific analysis revealed important differences. Femoral revisions were uncommon in our cohort and equivalent between MP and PS designs. However, PS implants demonstrated significantly higher tibial revision risk compared to MP designs. The higher revision rate observed in PS designs may reflect biomechanical differences between the two implant types. While PS designs control kinematics through a cam‐post mechanism, MP designs employ a medial ‘ball‐in‐socket’ articulation with unconstrained lateral compartment motion, generating different stress patterns on the tibial component [25].

Studies have demonstrated the biomechanical advantages of MP designs, with research showing that combining MP designs with kinematic alignment principles can better mimic native knee kinematics compared to mechanical alignment with traditional implant designs [9, 13, 25]. This biomechanical performance may contribute to the lower tibial revision rates observed in our study.

However, the clinical significance of these findings must be interpreted carefully, as the overall tibial revision rate of 0.36% remains very low. Furthermore, meta‐analyses comparing overall revision rates between MP and PS TKAs have found no statistically significant differences in general revision outcomes [12, 14], suggesting that while component‐specific differences may exist, overall implant survivorship appears comparable between the two designs.

Several limitations must be acknowledged in this registry‐based study. As with all registry analyses, we lacked detailed clinical and radiographic parameters. The relatively low event rates (2.29% for patellar resurfacing, 0.12% for femoral revision and 0.36% for tibial revision) limited statistical power for complex multivariate analyses and subgroup comparisons. Additionally, our findings are limited to cemented, cruciate‐sacrificing MP and PS TKAs. Our analysis compared MP and PS as broad design categories without accounting for implant‐level variation in patellofemoral design characteristics such as trochlear geometry [6, 29]. Furthermore, the indication for primary patellar resurfacing—whether systematic, selective, or never performed—is not recorded in the SIRIS registry and varies substantially between surgeons and institutions [5]. Additional unmeasured confounders include alignment philosophy and its influence on patellofemoral kinematics, patellar tilt, surgical approach and restoration of anterior femoral offset [19]. Despite these limitations, our study provides valuable real‐world evidence comparing MP and PS TKA designs in a large population with standardized follow‐up procedures.

## CONCLUSION

This registry analysis found no significant difference in secondary patellar resurfacing rates between MP and PS designs in primary TKA. However, PS designs are associated with increased tibial revision risk compared to MP designs. While absolute revision rates remain low for both designs, these component‐specific differences may reflect underlying biomechanical disparities between implant types.

## AUTHOR CONTRIBUTIONS

Patrick Pflüger carried out the data curation, statistical analysis and drafted the manuscript. Alberto Pedrazzini helped with data acquisition and processing. Lukas Jud, Lazaros Vlachopoulos and Sandro F. Fucentese participated in its design and helped draft the manuscript. All authors read and approved the final manuscript.

## CONFLICT OF INTEREST STATEMENT

Sandro F. Fucentese is a consultant for Medacta SA (Switzerland), Smith & Nephew (United Kingdom), Zimmer Biomet and Karl Storz SE & Co. KG (Germany). The other authors declare no conflicts of interest.

## ETHICS STATEMENT

This analysis utilizes data from the national knee joint registry. The data provided for the analysis were de‐identified.

## Data Availability

The data that support the findings of this study are available from the corresponding author upon reasonable request.
